# Perioperative Outcomes of Cemented vs Cementless Total Hip Arthroplasty: A National Inpatient Sample Study of 81,668 Elective Procedures

**DOI:** 10.3390/jcm15093292

**Published:** 2026-04-25

**Authors:** Assil Mahamid, Mustafa Yassin, Basil Habiballa, Mohanad Natsheh, Hamza Murad, Khaled Qassem, Dror Robinson, Barak Haviv, Ali Yassin, Muhammad Khatib

**Affiliations:** 1 Department of Orthopedics, Hasharon Hospital, Rabin Medical Center, Affiliated to Tel Aviv University, Tel Aviv 6997801, Israeldror61@gmail.com (D.R.);; 2 Gray Faculty of Medical & Health Sciences, Tel Aviv University, Tel Aviv 6997801, Israel; 3 Department of Orthopedics, Hillel Yaffe Medical Center, Hadera 3820302, Israel

**Keywords:** total hip arthroplasty, cemented fixation, cementless fixation, national inpatient sample, periprosthetic fracture, blood transfusion, acute kidney injury, postoperative delirium, length of stay, healthcare utilization

## Abstract

**Background:** Cemented and cementless fixation techniques in total hip arthroplasty (THA) each present distinct biomechanical properties and perioperative risk profiles. While cementless fixation has gained increasing popularity, large-scale nationally representative comparisons of perioperative outcomes between cemented and cementless elective THA remain limited. This study aimed to compare complication rates, healthcare utilization, and temporal trends between cemented and cementless elective THA using the National Inpatient Sample. **Methods:** A retrospective cohort study was conducted using the National Inpatient Sample database from 2016 to 2021. Adult patients undergoing elective primary total hip arthroplasty were identified using ICD-10-PCS codes and categorized into cemented and cementless fixation groups. Patient demographics, comorbidities, indications, postoperative complications, length of stay, hospital charges, and in-hospital mortality were compared. Multivariate logistic regression analysis was performed to evaluate the independent association between fixation type and postoperative complications while adjusting for demographic, clinical, and hospital-level variables. **Results:** A total of 81,668 elective THAs were identified, including 40,290 cemented (49.33%) and 41,378 cementless (50.67%) procedures. Cemented THA was associated with a shorter length of stay (2.09 ± 1.88 vs. 2.26 ± 2.47 days, *p* < 0.001) and lower total hospital charges ($65,584.53 ± 48,797.21 vs. $72,186.84 ± 49,860.20, *p* < 0.001). Unadjusted analyses demonstrated higher rates of acute kidney injury and sepsis in the cementless group. After multivariate adjustment, cemented fixation was associated with lower odds of acute kidney injury (OR 0.87, 95% CI 0.79–0.96, *p* = 0.004). However, cemented THA was associated with higher odds of postoperative delirium (OR 1.20, 95% CI 1.02–1.42, *p* = 0.030), blood transfusion (OR 1.27, 95% CI 1.17–1.37, *p* < 0.001), and periprosthetic fracture (OR 1.32, 95% CI 1.02–1.71, *p* = 0.035). Rates of myocardial infarction, pneumonia, venous thromboembolism, urinary tract infection, and in-hospital mortality were similar between groups. Temporal analysis demonstrated comparable utilization trends, with a decline in elective procedures during 2020–2021. **Conclusions:** In this nationwide analysis, cemented total hip arthroplasty was associated with lower risk of acute kidney injury, shorter length of stay, and lower hospital charges, but higher odds of postoperative delirium, blood transfusion, and periprosthetic fracture compared with cementless fixation. These findings highlight distinct perioperative risk profiles between fixation strategies and may assist surgeons in individualized decision-making for elective total hip arthroplasty.

## 1. Introduction

Total hip arthroplasty (THA) is one of the most commonly performed elective surgical procedures in the United States, with annual volumes projected to reach 572,000 by 2030 [1]. Described as the “operation of the century,” THA reliably restores function and significantly improves quality of life across a broad patient population [2]. The predominant indication is end-stage hip osteoarthritis, a degenerative condition driven by progressive articular cartilage loss and subchondral remodeling [3]. Additional indications include osteonecrosis of the femoral head, developmental dysplasia of the hip (DDH), inflammatory arthropathies, and femoroacetabular impingement (FAI). When non-operative treatment fails, THA remains the definitive intervention.

A central technical decision in THA is the choice between cemented and cementless implant fixation. Cemented fixation, pioneered by Charnley in the 1960s, uses polymethylmethacrylate (PMMA) bone cement to achieve immediate mechanical stability and has demonstrated excellent long-term survivorship, particularly in elderly and lower-demand patients [4]. Cementless fixation, developed in the 1980s, relies on press-fit and subsequent osseointegration, avoiding cement-related complications while offering advantages in revision settings [5]. Registry data from Sweden and Australia demonstrate that cemented constructs continue to predominate in many older patient populations [6], yet the United States has experienced a progressive and substantial shift toward cementless THA across virtually all age groups, a trend driven by surgeon training patterns, evolving implant technology, and mid-term survivorship data [7].

Each fixation method carries a distinct perioperative risk profile. Cemented THA is associated with bone cement implantation syndrome (BCIS), a serious cardiovascular complication resulting from systemic fat, marrow, and monomer embolization during femoral canal pressurization, with greatest risk in patients with underlying cardiopulmonary disease [8]. Cementless THA, conversely, is associated with a higher risk of intraoperative and early periprosthetic femoral fracture due to the mechanical demands of press-fit impaction, particularly in osteoporotic bone [9]. Abdel et al. (2016) demonstrated that intraoperative fractures occurred in 3.0% of cementless stems compared with only 0.23% of cemented stems in a 40-year single-center series of 32,644 primary THAs [10]. Systemically, differences in rates of acute kidney injury (AKI), sepsis, venous thromboembolism, and blood transfusion between fixation groups remain incompletely characterized at the population level [11].

Despite growing utilization of cementless fixation, large-scale nationally representative data comparing perioperative outcomes between cemented and cementless elective THA in a U.S. administrative database context remain limited. Prior studies have frequently been limited by single-center referral bias, restricted sample sizes, or registry data that may not reflect the full breadth of practice patterns across the U.S. healthcare system [12]. The National Inpatient Sample (NIS), maintained by the Healthcare Cost and Utilization Project (HCUP), captures approximately 20% of all domestic hospitalizations and enables nationally weighted outcome estimates across a socioeconomically diverse patient population [13]. To date, no NIS-based analysis has comprehensively compared perioperative complications, hospital resource utilization, and temporal fixation trends between cemented and cementless elective THA across the 2016–2021 period.

Therefore, the objective of this retrospective cohort study was to compare perioperative outcomes, complication profiles, length of stay, total hospital charges, and in-hospital mortality between patients undergoing cemented versus cementless elective THA using the NIS from 2016 to 2021, and to identify independent predictors of adverse outcomes through multivariate logistic regression adjusted for patient demographics, comorbidity burden, and hospital-level characteristics. To our knowledge, no prior study using a contemporary nationwide U.S. administrative database has comprehensively compared perioperative complications, healthcare utilization, and temporal trends between cemented and cementless elective total hip arthroplasty.

## 2. Materials and Methods

### 2.1. Data Source

This retrospective cohort study utilized data from the National Inpatient Sample (NIS), the largest publicly available all-payer inpatient healthcare database in the United States, developed by the Agency for Healthcare Research and Quality (AHRQ) as part of the Healthcare Cost and Utilization Project (HCUP). The NIS approximates a 20% stratified sample of U.S. community hospital discharges and provides discharge-level weights that allow generation of national estimates. The study period included hospitalizations from 1 January 2016, through 31 December 2021. Each record in the NIS represents a single hospitalization rather than an individual patient, and analyses were performed using discharge weights to produce nationally representative estimates.

### 2.2. Study Population

Adult patients aged 18 years and older who underwent elective primary total hip arthroplasty were identified using International Classification of Diseases, Tenth Revision, Procedure Coding System (ICD-10-PCS) codes. Cemented total hip arthroplasty procedures were identified using codes 0SR90JA and 0SRB0JA, corresponding to right and left hip replacement with a cemented synthetic substitute. Cementless total hip arthroplasty procedures were identified using codes 0SR90JZ and 0SRB0JZ, corresponding to right and left hip replacement with a cementless synthetic substitute.

To ensure inclusion of primary arthroplasty procedures, workflow filtering was performed using I10_PR1 procedure identifiers including 0SR90K9, 0SRB0K9, 0SR90J9, and 0SRB0J9. Only elective admissions were included in the final cohort using the elective admission variable (ELECTIVE = 1). Hospitalizations associated with non-elective admissions, trauma-related arthroplasty, or revision procedures were excluded. After application of all inclusion and exclusion criteria, a total of 102,422 total hip arthroplasty procedures were identified. Among these, 81,668 were elective total hip arthroplasties. The final analytic cohort consisted of 40,290 cemented elective total hip arthroplasties and 41,378 cementless elective total hip arthroplasties.

### 2.3. Variables and Outcomes

Patient-level variables extracted from the NIS included age, sex, race, primary payer, median household income quartile, and Elixhauser comorbidities. Hospital-level variables included hospital region, bed size, and teaching status. The primary objective of this study was to compare inpatient outcomes between cemented and cementless total hip arthroplasty. Primary outcomes included in-hospital mortality, length of stay, and total hospital charges. Secondary outcomes included postoperative complications such as acute renal failure, respiratory complications, myocardial infarction, venous thromboembolism including deep vein thrombosis and pulmonary embolism, postoperative infection, blood transfusion, and discharge disposition. Prolonged length of stay and high hospital charges were defined as values above the 75th percentile of the study population. This study evaluated outcomes limited to the index hospitalization, and therefore does not assess long-term postoperative outcomes or implant survivorship.

### 2.4. Statistical Analysis

All statistical analyses were performed using R statistical software (version 4.3.2). Survey weights provided by the HCUP National Inpatient Sample were applied to account for the complex sampling design and to generate nationally representative estimates. Continuous variables were reported as mean ± standard deviation or median ± standard deviation as appropriate, while categorical variables were expressed as percentages. Baseline demographic characteristics, hospital variables, indications for surgery, and comorbidities were compared between cemented and cementless total hip arthroplasty groups using the chi-square test for categorical variables and Student’s *t*-test for continuous variables. Unadjusted comparisons were performed for inpatient outcomes including acute kidney injury, myocardial infarction, pneumonia, sepsis, venous thromboembolism, urinary tract infection, postoperative delirium, blood transfusion, and periprosthetic fracture. Additional comparisons included length of stay, total hospital charges, and in-hospital mortality.

To evaluate the independent association between fixation type and postoperative outcomes, multivariate logistic regression models were constructed. Cemented total hip arthroplasty served as the reference group. The regression models were adjusted for demographic and hospital-level variables including age, sex, race, primary payer, hospital bed size, admission timing (weekend versus weekday), and relevant comorbidities including hypertension, hyperlipidemia, obesity, diabetes, smoking status, chronic pulmonary disease, congestive heart failure, coagulopathy, and other Elixhauser comorbidity variables. Adjusted odds ratios with 95% confidence intervals were calculated for each postoperative complication. A two-sided *p*-value less than 0.05 was considered statistically significant.

### 2.5. Ethical Considerations

The NIS database contains fully de-identified publicly available data; therefore, institutional review board approval and informed consent were not required. The study was conducted in accordance with HCUP data use agreement recommendations for secondary analysis of administrative databases.

## 3. Results

**Figure 1:**
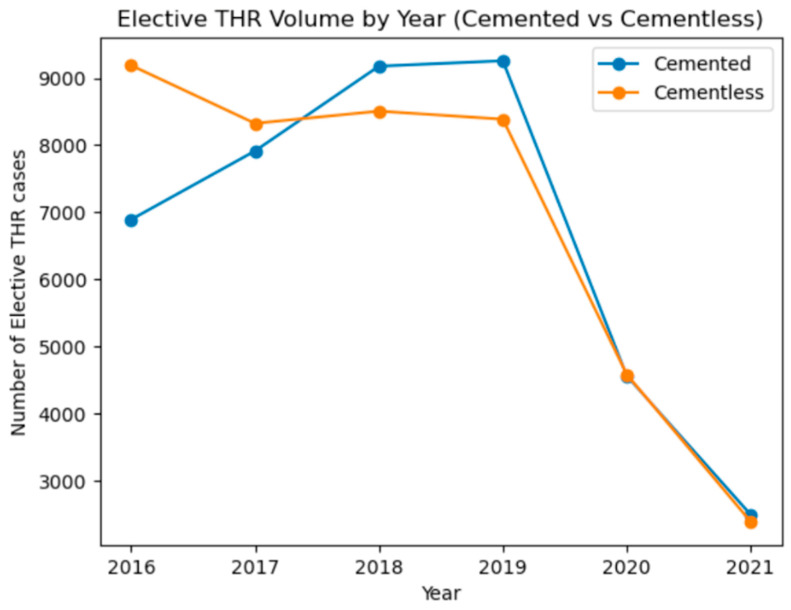
Temporal Trends in Elective Cemented and Cementless Total Hip Arthroplasty.

Temporal analysis demonstrated changing patterns between cemented and cementless total hip arthroplasty over the study period. Cementless procedures were more common in 2016 and 2017, whereas cemented fixation increased between 2017 and 2019 and exceeded cementless procedures during this period. Both cemented and cementless total hip arthroplasty volumes declined sharply in 2020, with a further reduction observed in 2021. Overall, the trends demonstrate comparable utilization of both fixation techniques with a marked decrease in elective total hip arthroplasty volume during the later years of the study period (Figure 1).

A total of 81,668 elective total hip arthroplasties were identified, including 40,290 cemented (49.33%) and 41,378 cementless (50.67%) procedures. Table 1 demonstrates that patients in the cemented group were slightly older than those in the cementless group (66.27 ± 11.12 vs. 66.02 ± 11.39 years, *p* < 0.001), while age group distribution and sex were similar between cohorts, with females comprising the majority in both groups (56.02% vs. 55.68%, *p* = 0.3). Racial distribution showed small but statistically significant differences, with a slightly higher proportion of White patients in the cemented group and higher proportions of Black and Hispanic patients in the cementless group (*p* < 0.001). Cemented procedures were more frequently performed in small hospitals, whereas cementless procedures were more common in medium-sized hospitals (*p* < 0.001). The cemented cohort had a shorter length of stay (2.09 ± 1.88 vs. 2.26 ± 2.47 days, *p* < 0.001) and lower total hospital charges ($65,584.53 ± 48,797.21 vs. $72,186.84 ± 49,860.2, *p* < 0.001). Weekend admissions were slightly more common in the cementless group (*p* = 0.002). Payer distribution and in-hospital mortality were similar between groups.

As shown in Table 2, osteoarthritis was the most common indication for total hip arthroplasty in both cohorts, accounting for 87.29% of cemented procedures and 85.56% of cementless procedures (*p* < 0.001). Osteonecrosis was slightly more frequent in the cementless group compared with the cemented group (2.72% vs. 2.49%, *p* = 0.039). Perthes disease was also more common among cementless arthroplasties, although the absolute incidence was low (0.07% vs. 0.03%, *p* = 0.02). There were no significant differences between groups in the rates of rheumatoid arthritis, developmental dysplasia of the hip, or femoroacetabular impingement.

Baseline comorbidities were largely comparable between cemented and cementless total hip arthroplasty cohorts. The most prevalent comorbidities in both groups were hypertension (52.93% vs. 52.84%), hyperlipidemia (33.16% vs. 33.35%), and gastroesophageal reflux disease (26.6% vs. 26.2%), without significant differences (Table 3). Former smoking history was slightly more common in the cemented cohort (23.8% vs. 22.7%, *p* = 0.0002), while obesity was also marginally higher among cemented procedures (14.2% vs. 13.6%, *p* = 0.01). Coagulopathy was more frequent in the cementless group (1.52% vs. 1.34%, *p* = 0.03). No significant differences were observed between groups for diabetes, hypothyroidism, chronic pulmonary disease, congestive heart failure, rheumatoid disease, chronic anemia, liver disease, metastatic cancer, neurological disorders, or major depressive disorder.

Postoperative complications were generally uncommon in both cohorts, as demonstrated in Table 4. Cementless total hip arthroplasty was associated with higher rates of acute kidney injury (2.59% vs. 2.09%, *p* < 0.001) and sepsis (0.16% vs. 0.09%, *p* = 0.002). Periprosthetic fracture was also more frequent in the cementless group (0.48% vs. 0.31%, *p* < 0.001). Rates of acute myocardial infarction, pneumonia, venous thromboembolism, urinary tract infection, postoperative delirium, and blood transfusion were similar between groups, with no statistically significant differences.

Cemented total hip arthroplasty was associated with a shorter length of stay compared with cementless procedures (2.09 ± 1.88 vs. 2.26 ± 2.47 days, *p* < 0.001). As demonstrated in Table 5, total hospital charges were significantly lower in the cemented cohort ($65,584.53 ± 48,797.21 vs. $72,186.84 ± 49,860.20, *p* < 0.001). In-hospital mortality was rare and comparable between groups, with no statistically significant difference observed (0.03% vs. 0.06%, *p* = 0.19).

**Figure 2:**
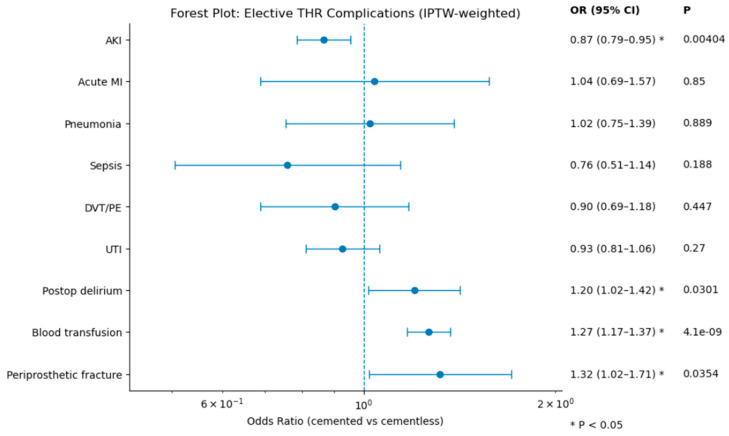
Multivariate Logistic Regression Analysis of Postoperative Complications Following Cemented vs. Cementless Total Hip Arthroplasty.

**Figure 3:**
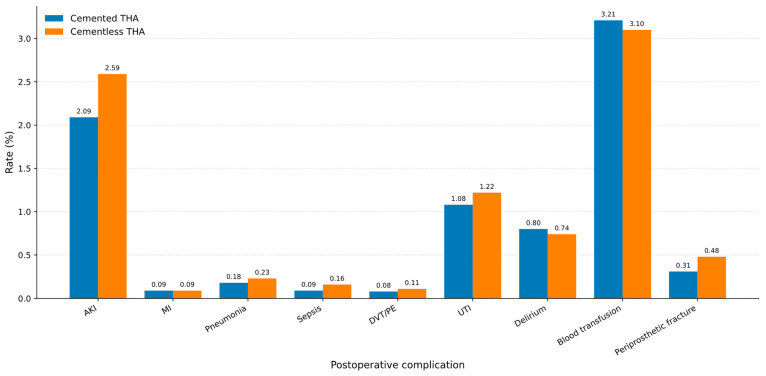
Unadjusted in-hospital complication rates after elective cemented versus cementless total hip arthroplasty. Bar chart comparing absolute rates of postoperative complications during the index hospitalization in patients undergoing cemented and cementless THA.

After multivariate adjustment, cemented total hip arthroplasty was associated with a lower risk of acute kidney injury compared with cementless fixation (OR 0.87, 95% CI 0.79–0.96, *p* = 0.004). No significant differences were observed between groups for acute myocardial infarction, pneumonia, sepsis, venous thromboembolism, or urinary tract infection. Cemented fixation was associated with higher odds of postoperative delirium (OR 1.20, 95% CI 1.02–1.42, *p* = 0.030), blood transfusion (OR 1.27, 95% CI 1.17–1.37, *p* < 0.001), and periprosthetic fracture (OR 1.32, 95% CI 1.02–1.71, *p* = 0.035). These findings are illustrated in the IPTW-weighted forest plot (Figure 2). In addition to the multivariate forest plot, absolute complication rates are presented graphically in Figure 3 to improve visualization of the unadjusted between-group differences. Consequently, the adjusted estimates should be interpreted as associative rather than causal, and may reflect both measured and unmeasured differences in patient selection. This pattern is consistent with confounding by indication, whereby fixation choice is influenced by patient-specific risk factors that also affect complication rates.

## 4. Discussion

In this large nationwide analysis of 81,668 elective total hip arthroplasties, cemented and cementless fixation were used at comparable rates. This study addresses an important gap in the literature by providing a large-scale, nationally representative comparison of perioperative outcomes and healthcare utilization between fixation strategies in contemporary practice. Cemented total hip arthroplasty was associated with a lower risk of acute kidney injury, shorter length of stay, and lower total hospital charges compared with cementless fixation. However, cemented procedures demonstrated higher odds of postoperative delirium, blood transfusion, and periprosthetic fracture. Rates of major cardiopulmonary and infectious complications, including myocardial infarction, pneumonia, sepsis, venous thromboembolism, and urinary tract infection, were similar between groups. Indications for surgery were largely comparable, with osteoarthritis representing the predominant diagnosis in both cohorts. Temporal analysis demonstrated comparable utilization trends between fixation types, with a marked decline in elective procedures during the later years of the study period. However, despite statistical significance, the absolute differences between groups were small and should be interpreted in the context of clinical relevance.

Although cemented total hip arthroplasty demonstrated lower intraoperative blood loss in prior studies, transfusion requirements have paradoxically been reported to be higher compared with cementless fixation. In a retrospective study of 1500 patients, fully cemented THA was associated with significantly lower blood loss than cementless THA (695 ± 74 mL vs. 957 ± 16 mL, *p* < 0.05), likely due to cement polymerization sealing cancellous bone and reducing intraoperative bleeding [1,14,15]. Earlier investigations similarly demonstrated greater postoperative blood loss in cementless procedures [15,16]. Despite this hemostatic effect, transfusion rates did not follow the same pattern, with reported transfusion rates ranging from 1.3% in cementless THA to 7.9% in cemented THA [1]. A matched-pair study further showed no overall difference in transfusion requirement, although transfused patients in the cemented group received larger volumes [14]. In the present study, cemented fixation was likewise associated with increased odds of blood transfusion compared with cementless THA (OR 1.27, 95% CI 1.17–1.37, *p* < 0.001), despite similar baseline comorbidity profiles between groups. These findings support prior literature suggesting that transfusion risk in cemented THA may not be directly related to intraoperative blood loss alone. Cement pressurization itself is not associated with increased bleeding but may introduce hemodynamic changes related to bone cement implantation syndrome (BCIS), which occurs in approximately 26% of cemented arthroplasties and is associated with embolization, reduced cardiac output, and increased pulmonary artery pressure [17,18]. Importantly, transfusion risk in THA appears to be primarily driven by patient-related factors rather than fixation technique. A 2025 meta-analysis of 424,158 patients identified preoperative anemia, advanced age, female sex, higher ASA class, low BMI, and increased perioperative bleeding as independent predictors of transfusion [19]. Machine learning models similarly identified demographic and hematologic variables as key determinants with excellent predictive performance [20]. The paradox of lower blood loss but higher transfusion rates in cemented THA is therefore likely explained by selection of older or more medically complex patients for cemented fixation, rather than a direct effect of cement use itself [1,21]. Recent high-level evidence from randomized controlled trials has suggested potential advantages of cemented fixation in specific patient populations. A systematic review and meta-analysis of randomized controlled trials in elderly patients with femoral neck fractures demonstrated improved health-related quality of life, reduced one-year mortality, and lower reoperation rates following cemented hemiarthroplasty compared with uncemented fixation [22]. These findings support the concept that cemented fixation may offer clinically meaningful benefits in selected populations. However, important differences in surgical indication, patient characteristics, and implant type must be considered, as hemiarthroplasty for fracture and elective total hip arthroplasty represent distinct clinical contexts. Therefore, the applicability of these findings to elective THA populations remains uncertain and should be interpreted with caution.

Postoperative delirium after total hip arthroplasty remains a multifactorial complication, and current evidence does not demonstrate that cemented fixation is independently associated with increased delirium risk compared with cementless techniques. Bone cement implantation syndrome (BCIS) may manifest postoperatively with hypoxia and confusion, which could theoretically contribute to delirium; however, the relationship between cement fixation and delirium is confounded by patient-related factors rather than fixation technique alone [8,23]. Systematic reviews applying Bradford–Hill criteria have found insufficient evidence to establish a causal relationship between cement use and BCIS-related complications, noting that similar intraoperative events may occur during cementless procedures [24]. The reported incidence of BCIS during cemented hip arthroplasty ranges from 15.4% to 46.7%, with severe BCIS associated with increased short-term mortality, and risk factors such as advanced age, higher ASA class, and renal impairment overlap substantially with known predictors of postoperative delirium [17,25]. Studies evaluating postoperative cognitive dysfunction after cemented versus cementless THA have shown mixed results. Elevated S100B levels have been observed after cemented THA, suggesting transient cerebral insult, but without corresponding differences in neuropsychological testing or long-term cognitive outcomes [26,27]. Furthermore, postoperative delirium appears more strongly associated with systemic inflammation, anesthetic exposure, and perioperative management factors rather than fixation method [28,29]. Meta-analyses report delirium incidence ranging from 3.0% to 13.6% after THA, with consistent risk factors including advanced age, dementia, stroke, psychiatric disease, diabetes, hypertension, renal disease, anemia, general anesthesia, and blood transfusion [30,31,32,33,34]. In the present study, cemented total hip arthroplasty was associated with a modestly increased risk of postoperative delirium compared with cementless fixation (OR 1.20, 95% CI 1.02–1.42, *p* = 0.030). This finding should be interpreted cautiously, as cement fixation is often preferentially used in older patients with reduced bone quality and higher comorbidity burden, characteristics that independently increase delirium risk. Indeed, major systematic reviews have not identified fixation method as an independent predictor of delirium following THA [17,21]. A recent propensity-matched analysis of patients older than 70 years demonstrated that cemented THA was associated with improved perioperative outcomes, including shorter length of stay, without increased delirium risk [21]. Therefore, the higher delirium rate observed in our analysis likely reflects residual confounding and patient selection rather than a direct causal effect of cement use.

Studies suggest that cemented total hip arthroplasty may reduce length of stay, particularly in older patients, and improve discharge-to-home rates compared with cementless fixation. Cemented prostheses may also allow for earlier mobilization due to immediate implant stability [21,35,36]. In patients aged 70–79 years, cemented THA has been associated with a significantly shorter length of stay compared with cementless fixation (2.2 vs 2.6 days, *p* = 0.017), with an even greater difference observed in patients aged ≥ 80 years (3.0 vs 3.4 days, *p* = 0.041) [21]. A Medicare-based analysis similarly demonstrated trends toward shorter hospitalization with cemented fixation, although this did not remain significant after adjustment for baseline differences [35]. These findings are consistent with the results of the present study, in which cemented THA was associated with a shorter length of stay compared with cementless fixation (2.09 ± 1.88 vs 2.26 ± 2.47 days, *p* < 0.001). The shorter hospitalization observed with cemented fixation may be related to improved early stability and facilitated mobilization.

This study has several important limitations that should be acknowledged. First, the analysis was based on the National Inpatient Sample, an administrative database that relies on ICD-10 coding, which may be subject to miscoding or underreporting of diagnoses and complications [37,38,39]. The database captures only inpatient hospitalizations and does not include postoperative follow-up data, readmissions, outpatient complications, or long-term outcomes, thereby limiting assessment of implant survivorship, late periprosthetic fracture, and revision risk following cemented versus cementless fixation. Second, the retrospective observational design precludes establishing causality between fixation method and postoperative outcomes. Third, several clinically relevant variables that may influence fixation choice and complications are not available in the NIS, including bone quality, femoral morphology, surgical approach, implant design, cementing technique, surgeon experience, operative time, and intraoperative blood loss. These unmeasured confounders may partially explain the observed differences in transfusion, delirium, and periprosthetic fracture rates. Importantly, because these critical variables are not captured, the observed associations between fixation method and perioperative outcomes should be interpreted as non-causal and may reflect underlying patient selection and surgical decision-making rather than the independent effect of fixation strategy. Fourth, complications were captured only during the index hospitalization, which likely underestimates events such as infection, thromboembolism, and delayed periprosthetic fracture. Additionally, functional outcomes, discharge mobility, and patient-reported outcomes are not available in the dataset. Accordingly, the findings of this study are limited to in-hospital perioperative outcomes, and no conclusions can be drawn regarding longer-term complications or implant survivorship. Finally, the study period included the COVID-19 pandemic years, which may have influenced elective arthroplasty volume, patient selection, and perioperative resource utilization. Despite these limitations, the large sample size and nationally representative weighting strengthen the generalizability of the findings.

The present study also has several strengths. To our knowledge, this represents one of the largest contemporary nationwide comparisons of cemented and cementless total hip arthroplasty, including more than 81,000 elective procedures. The use of the National Inpatient Sample enabled evaluation across diverse hospital settings, geographic regions, and patient populations, improving external validity. The study provides a comprehensive comparison of demographic characteristics, indications, comorbidities, perioperative complications, and healthcare utilization outcomes between fixation strategies. Additionally, the use of multivariate logistic regression modeling allowed adjustment for multiple demographic, hospital, and comorbidity-related confounders, improving the robustness of the observed associations. The inclusion of cost analysis and temporal utilization trends further enhances the clinical and health-systems relevance of the findings. Collectively, these strengths support the reliability of the results and provide meaningful insight into contemporary outcomes of cemented versus cementless total hip arthroplasty.

## 5. Conclusions

In this nationwide analysis of elective total hip arthroplasty, cemented and cementless fixation demonstrated distinct perioperative risk profiles. Cemented fixation was associated with shorter length of stay, lower hospital charges, and reduced odds of acute kidney injury. However, it was also associated with higher odds of blood transfusion, postoperative delirium, and periprosthetic fracture. Other major cardiopulmonary and infectious complications, as well as in-hospital mortality, were comparable between groups. These findings suggest that fixation strategy influences selected perioperative outcomes and resource utilization, and should be individualized according to patient characteristics and comorbidity burden. These findings should therefore be interpreted with caution, as unmeasured clinical and technical factors may significantly influence both treatment selection and observed outcomes. Further prospective studies with longitudinal follow-up are required to evaluate the impact of fixation method on long-term outcomes.

## Figures and Tables

**Table 1:** Baseline Demographic and Hospital Characteristics of Patients Undergoing Elective Cemented vs. Cementless Total Hip Arthroplasty.

Variable	Cemented (n = 40,290)	Cementless (n = 41,378)	*p* Value
**Total incidence (%)**	49.33	50.67	—
**Age, mean ± SD (years)**	66.27 ± 11.12	66.02 ± 11.39	<0.001
**Age group (%)**			
18–44	3.31	3.76	
45–64	37.43	38.01	
65–74	35.94	35.19	
≥75	23.32	23.03	
**Sex (%)**			0.30
Female	56.02	55.68	
Male	43.98	44.32	
**Race (%)**			
White	84.91	83.21	<0.001
Black	8.30	9.30	<0.001
Hispanic	3.71	4.32	<0.001
Asian or Pacific Islander	0.95	0.77	0.007
Native American	0.32	0.25	0.06
Other	1.77	2.12	<0.001
**Hospital bed size (%)**			
Small	30.28	28.70	<0.001
Medium	28.59	31.06	<0.001
Large	41.12	40.24	0.01
**Length of stay, mean ± SD (days)**	2.09 ± 1.88	2.26 ± 2.47	<0.001
**Admission timing (%)**			0.002
Weekend	0.39	0.54	
Weekday	99.61	99.46	
**Primary payer (%)**			
Medicare	57.06	56.51	0.10
Medicaid	5.33	5.55	0.10
Private insurance	34.19	34.30	0.70
Self-pay	0.72	0.77	0.40
No charge	0.07	0.09	0.30
Other	2.63	2.79	0.10
**Total hospital charges, mean ± SD ($)**	65,584.53 ± 48,797.21	72,186.84 ± 49,860.20	<0.001
**In-hospital mortality (%)**	0.03	0.06	0.19

**Table 2:** Indications for Elective Cemented vs. Cementless Total Hip Arthroplasty.

Indication	Cemented (n = 40,290)	Cementless (n = 41,378)	*p* Value
Osteoarthritis (%)	87.29	85.56	<0.001
Osteonecrosis (%)	2.49	2.72	0.039
Rheumatoid arthritis (%)	0.17	0.16	0.74
Developmental dysplasia of the hip (%)	0.11	0.11	0.90
Perthes disease (%)	0.03	0.07	0.02
Femoroacetabular impingement (%)	0.01	0.01	0.90

**Table 3:** Comorbidities in Patients Undergoing Elective Cemented vs. Cementless Total Hip Arthroplasty.

Comorbidity	Cemented (n = 40,290)	Cementless (n = 41,378)	*p* Value
Essential hypertension (%)	52.93	52.84	0.79
Hyperlipidemia (%)	33.16	33.35	0.55
Gastroesophageal reflux disease (%)	26.60	26.20	0.10
Former smoker (%)	23.80	22.70	0.0002
Hypothyroidism (%)	14.30	14.10	0.52
Obesity (%)	14.20	13.60	0.01
Diabetes mellitus (%)	14.86	15.15	0.25
Chronic anemia (%)	2.10	2.06	0.70
Rheumatoid disease (%)	2.84	2.82	0.80
Congestive heart failure (%)	3.32	3.35	0.70
Chronic pulmonary disease (%)	13.70	13.92	0.30
Coagulopathy (%)	1.34	1.52	0.03
Liver disease (%)	0.72	0.75	0.59
Metastatic cancer (%)	0.14	0.15	0.59
Neurological disorders (%)	1.90	1.80	0.28
Major depressive disorder (%)	11.50	11.80	

**Table 4:** Postoperative Complications Following Cemented vs. Cementless Total Hip Arthroplasty.

Complication	Cemented (n = 40,290)	Cementless (n = 41,378)	*p* Value
Acute kidney injury (%)	842 (2.09)	1069 (2.59)	<0.001
Acute myocardial infarction (%)	36 (0.09)	33 (0.09)	0.555
Pneumonia (%)	73 (0.18)	95 (0.23)	0.17
Sepsis (%)	36 (0.09)	66 (0.16)	0.002
DVT/PE (%)	32 (0.08)	46 (0.11)	0.14
Urinary tract infection (%)	435 (1.08)	505 (1.22)	0.06
Postoperative delirium (%)	322 (0.80)	306 (0.74)	0.309
Blood transfusion (%)	3.21	3.10	0.40
Periprosthetic fracture (%)	126 (0.31)	200 (0.48) *	<0.001

* Percentage calculated from cohort size.

**Table 5:** In-Hospital Outcomes Following Cemented vs. Cementless Total Hip Arthroplasty.

Outcome	Cemented (n = 40,290)	Cementless (n = 41,378)	*p* Value
Length of stay, mean ± SD (days)	2.09 ± 1.88	2.26 ± 2.47	<0.001
Total hospital charges, mean ± SD ($)	65,584.53 ± 48,797.21	72,186.84 ± 49,860.20	<0.001
In-hospital mortality (%)	0.03	0.06	0.19

## Data Availability

The data that support the findings of this study are available from the Healthcare Cost and Utilization Project (HCUP) National Inpatient Sample (NIS). These data are publicly available for purchase from the Agency for Healthcare Research and Quality (AHRQ) (https://www.hcup-us.ahrq.gov/nisoverview.jsp (Accessed on 1 December 2025)). The authors are not permitted to share the raw data directly due to HCUP data use agreements.

## Abbreviations

The following abbreviations are used in this manuscript:

AHRQ	Agency for Healthcare Research and Quality
AKI	Acute kidney injury
ASA	American Society of Anesthesiologists
BCIS	Bone cement implantation syndrome
CI	Confidence interval
DDH	Developmental dysplasia of the hip
DVT	Deep vein thrombosis
ELECTIVE	Elective admission variable
FAI	Femoroacetular impingement
HCUP	Healthcare Cost and Utilization Project
ICD-10-PCS	International Classification of Diseases, Tenth Revision, Procedure Coding System
IPTW	Inverse probability of treatment weighting
LOS	Length of stay
MI	Myocardial infarction
NIS	National Inpatient Sample
OR	Odds ratio
PE	Pulmonary embolism
PMMA	Polymethylmethacrylate
SD	Standard deviation
THA	Total hip arthroplasty
UTI	Urinary tract infection
